# A Large Mature Teratoma in a Postmenopausal Women

**DOI:** 10.1155/crog/6630801

**Published:** 2026-07-23

**Authors:** Shumarova Svetlana, Karamisheva Vesela

**Affiliations:** ^1^ Department of Surgery, University Hospital “Aleksandrovska” Sofia, Bulgaria, Medical University, Sofia, Bulgaria, mu-sofia.bg; ^2^ Department of Obstetrics and Gynecology, Faculty of Medicine, Medical University of Sofia, Bulgaria, SBALAG “Maichin Dom”, Sofia, Bulgaria, mu-sofia.bg

**Keywords:** germ cell tumor, mature teratoma, ovary, teratoma

## Abstract

Mature cystic teratomas arise from three germ lines: ectoderm, mesoderm, and endoderm. They have a certain malignant potential, which is a diagnostic and therapeutic challenge. They occur very often in young women, but there are a significant number of postmenopausal cases where the risk of malignancy is greater. Symptoms may be nonspecific, but in the event of complications, an acute condition may develop, threatening the patient′s life. Treatment depends on the risk of malignancy, age, and presence of complications. We present a case of a postmenopausal woman with a large mature teratoma of the left ovary involving elements from all three germ lines. A laparotomy and hysterectomy with bilateral salpingo‐oophorectomy were performed, considering the patient′s age and risk of malignancy. Given the risk of complications and malignancy in patients with mature teratoma, early detection, correct diagnosis, and therapeutic management are crucial for a higher chance of a successful outcome.

## 1. Introduction

Ovarian teratomas arise from ovarian germ cells (GCT) and are of three types: immature teratoma, mature teratoma, and monodermal teratoma. Mature teratoma occurs mostly in young female patients with a mean age of about 38.0 ± 13.3 years [1] and only in 5% of postmenopausal women [2]. Although they are benign, transformation into a malignant variant is possible in 0.3% [1]. They are very often asymptomatic and are found accidentally during a gynecological or other examination. When symptoms are present, they most often include pain, a feeling of abdominal distension, or others resulting from the compression of neighboring organs. They can mimic an acute condition in the presence of complications such as rupture [3, 4] and torsion [5], which often require the involvement of a multidisciplinary team. Computed tomography (CT) and magnetic resonance imaging (MRI) have the highest specificity and sensitivity for diagnosis. We present a case of a 70‐year‐old postmenopausal woman with a large mature teratoma involving elements from all three germ lines.

## 2. Case Presentation

**Figure 1:**
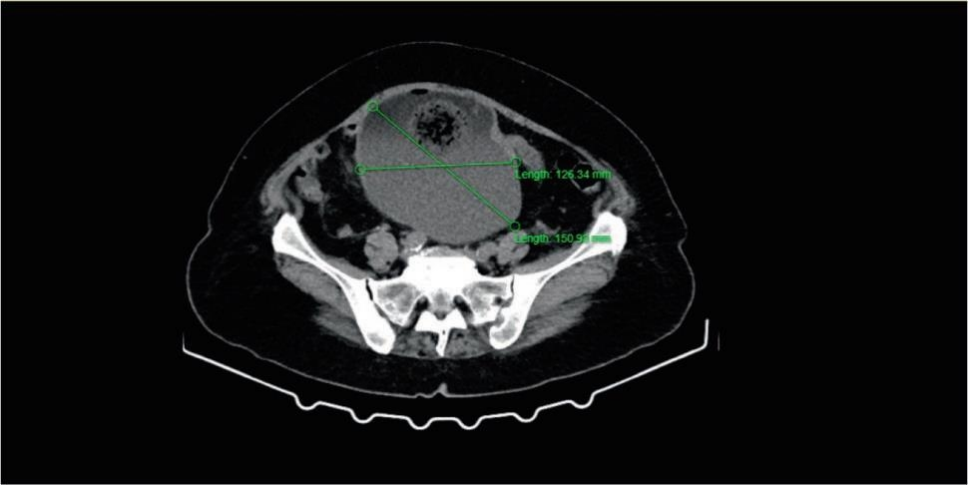
CT image of a mature teratoma on the left ovary.

**Figure 2:**
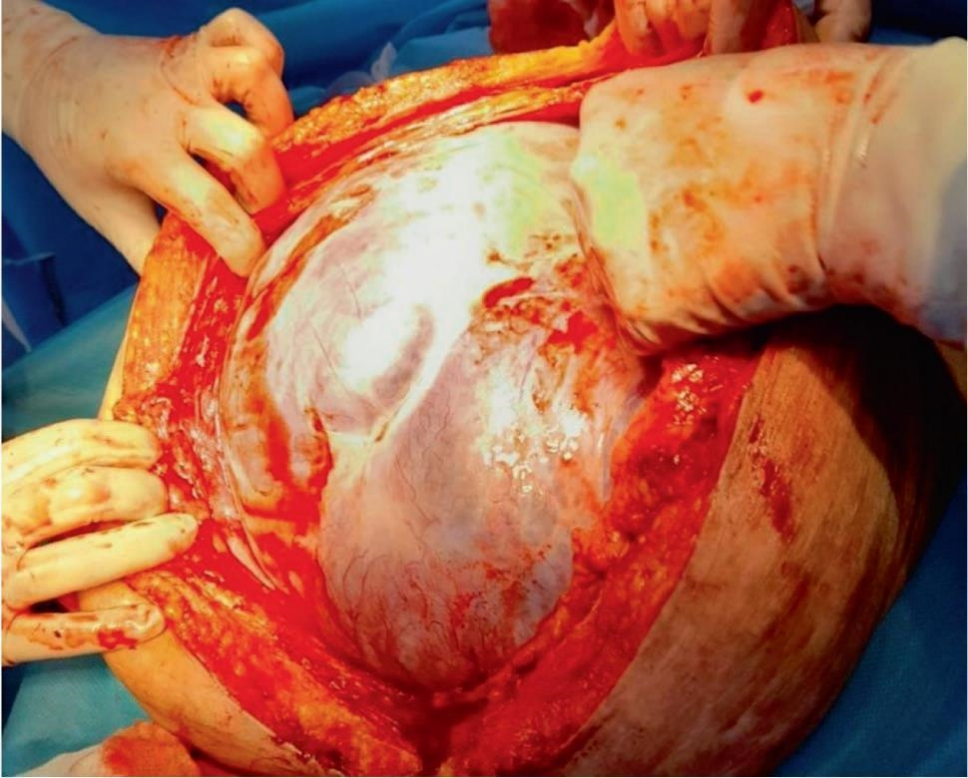
Macroscopic view of a mature teratoma on the left ovary.

**Figure 3:**
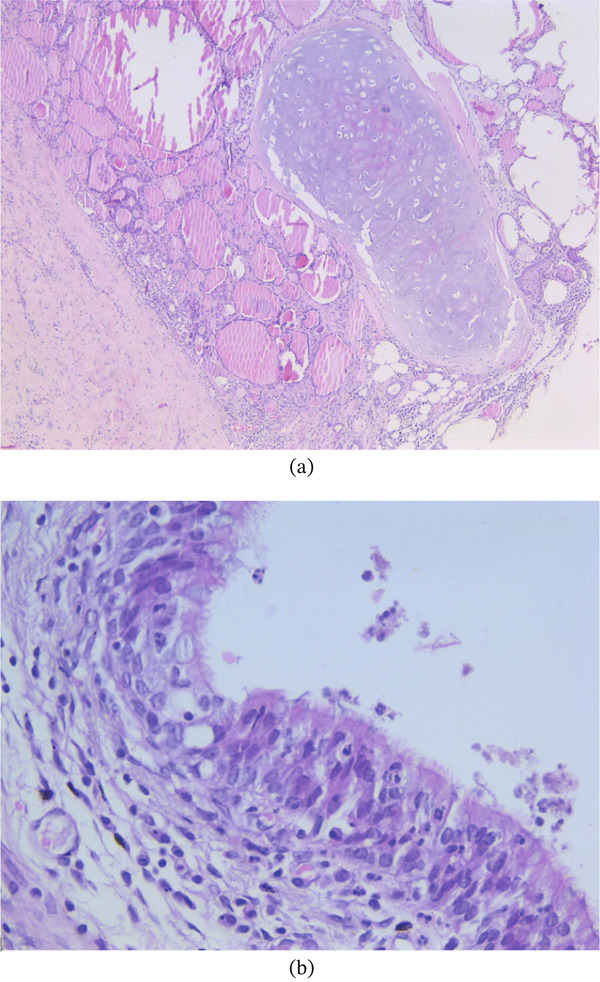
(a) H&E‐stained histological section showing cartilaginous tissue and surrounding skeletal muscle, thyroid tissue. Scale bar = 500 *μ*m. (b) High‐power H&E‐stained histological image showing respiratory epithelial lining and stromal inflammatory infiltrate. Scale bar = 50 *μ*m.

A 70‐year‐old female with complaints of abdominal distention and fatigue was admitted to a surgery clinic, where a large abdominal tumor formation with the appearance of a solid with cystic components was found on an abdominal ultrasound. A CT scan of the abdomen showed cystic formation measuring about 150.9 mm/d with fluid levels (Figure 1). There is suggestive evidence of a fistula between the sigmoid colon and the formation. The conclusion was suggestive of an ovarian teratoma, probably arising from the left ovary, from the laboratory indicators with data on leukocytosis. The results of studied tumor markers CA 125, CEA, and CA 19‐9 were normal. The patient reported one birth in a normal pregnancy, without any complications. She receives regular therapy for arterial hypertension. In a team with a gynecologist, laparotomy and hysterectomy with bilateral salpingo‐oophorectomy were performed considering the age and risk of malignancy (Figure 2). The decision to perform a laparotomy was made due to the increased risk of iatrogenic injury during trocar placement in laparoscopic surgery, given the large size of the tumor. The suspicion of a fistula was ruled out intraoperatively after release of the tumor adhesion to the sigmoid colon. Histological examination revealed a mature teratoma of the left ovary containing squamous epithelium, hair follicles, muscle, cartilage, and thyroid tissue (Figure 3a,b). Uterine adenomyosis and intramural hyalinized leiomyoma were also found. A frozen section has not been performed. The patient recovered without surgical complications and was discharged on the sixth postoperative day. Six months after the surgery, the patient was admitted to the surgical clinic with clinical evidence of acute cholecystitis, after which a laparoscopic cholecystectomy was performed. No pathological changes related to the previous surgery were found.

## 3. Discussion

Mature cystic teratomas involve elements of the three germ layers—mesoderm, endoderm, and exoderm—and can transform malignantly from any one of these three germ lines. The most common malignant transformation of teratoma is squamous cell carcinoma [6, 7] originating from the ectoderm, but rare cases of transformation into melanoma [8], carcinoid tumor [9], and mucinous intestinal adenocarcinoma [10] have also been described. Malignant transformation has been described in patients both under 45 years of age [7, 10] and in patients over 50 years of age [8, 9]. Since it is obvious that the transformation into a malignant process can also occur in young women, a detailed and exact diagnosis with imaging methods such as CT and MRI is necessary.

Centromeric indicators and enzymatic analyses are widely used to assess the karyotype of tumor tissues in mature cystic teratomas, which most commonly exhibit a normal female karyotype (46,XX) [11]. The analyses obtained by both methods are similar and demonstrate homozygosity in teratomas and heterozygosity in the host cells. Teratomas frequently differ from the host tissue, and in some cases, chromosomal abnormalities such as triploidy, tetraploidy, and mosaicism have been reported [11].

Some authors report elevated values of some tumor markers in the presence of malignant transformation such as CEA [7, 12], CA 125 [7, 12], and CA 19‐9 [12], but others report normal values of CEA and CA 125 [9]. There is still insufficient accumulated data and analysis on the relationship of tumor markers with cases of malignant transformation in teratomas and their prognostic value.

Often, patients have nonspecific complaints, and the findings are found incidentally most often during an ultrasound examination. Clinical complaints in uncomplicated forms of teratoma usually include pain of a different nature [7–9], constipation, frequent urination, and loss of appetite [7]. In the presence of a complication such as rupture or torsion, patients usually report prolonged and increasing pain in the abdomen [3, 4], as well as nausea and vomiting [12].

The management of a mature teratoma is influenced by the risk of malignancy, the age of the patient, and the requirement for fertility reserve [11]. Surgical removal is beneficial and includes ovary‐sparing surgery (OSS) or oophorectomy via laparotomy or laparoscopy. With its widespread entry into surgical practice, laparoscopy has become the gold standard for operative treatment due to a number of advantages such as faster recovery, less postoperative pain, and shorter hospital stay. It also has its drawbacks, such as the risk of rupture of the cyst during placement of the trocars, which would lead to spillage and chemical peritonitis with the formation of adhesion [3]. Cong et al. [11] proposed a treatment algorithm in the presence of malignant transformation, including three options: surgical treatment, chemotherapy, or targeted therapy. Benign cystic teratomas are associated with an excellent prognosis following surgical treatment, although recurrence has been reported between 2 and 10 years after surgery [13]. In the presence of malignant transformation, outcomes are variable and depend on the tumor stage, growth pattern, and vascular invasion [13].

## 4. Conclusion

This clinical case demonstrates that, in postmenopausal patients, a mature ovarian teratoma should not be regarded as a routine benign finding. Patient age and the potential for malignant transformation justify an oncologically oriented surgical approach, making this case a valuable contribution to optimizing surgical strategy in this specific patient population.

## Funding

No funding was received for this manuscript.

## Consent

Informed written consent was achieved from the patient.

## Conflicts of Interest

The authors declare no conflicts of interest.

## Data Availability

Data sharing is not applicable to this article as no datasets were generated or analyzed during the current study.
